# Feasibility of CO_2_ mitigation and carbohydrate production by microalga *Scenedesmus obliquus* CNW-N used for bioethanol fermentation under outdoor conditions: effects of seasonal changes

**DOI:** 10.1186/s13068-017-0712-5

**Published:** 2017-01-31

**Authors:** Shih-Hsin Ho, Yi-Di Chen, Ching-Yu Chang, Yen-Ying Lai, Chun-Yen Chen, Akihiko Kondo, Nan-Qi Ren, Jo-Shu Chang

**Affiliations:** 1 State Key Laboratory of Urban Water Resource and Environment, School of Municipal and Environmental Engineering, Harbin Institute Technology, Harbin, People’s Republic of China; 2 Department of Chemical Engineering, National Cheng Kung University, Tainan, Taiwan; 3 Center for Bioscience and Biotechnology, National Cheng Kung University, Tainan, Taiwan; 4 Department of Chemical Science and Engineering, Graduate School of Engineering, Kobe University, 1-1 Rokkodai, Nada-ku, Kobe, 657-8501 Japan; 5 Core Research for Evolutional Science and Technology, Japan Science and Technology Agency, 3-5 Sanbancho, Chiyoda-ku, Tokyo, 102-0075 Japan; 6 Biomass Engineering Program, RIKEN, 1-7-22 Suehiro, Tsurumi-ku, Yokohama, 230-0045 Japan; 7 Research Center for Energy Technology and Strategy, National Cheng Kung University, Tainan, Taiwan

**Keywords:** CO_2_ fixation, Bioethanol production, Microalgae, Carbohydrate, Outdoor cultivation, Seasonal changes

## Abstract

**Background:**

Although outdoor cultivation systems have been widely used for mass production of microalgae at a relatively low cost, there are still limited efforts on outdoor cultivation of carbohydrate-rich microalgae that were further used as feedstock for fermentative bioethanol production. In particular, the effects of seasonal changes on cell growth, CO_2_ fixation, and carbohydrate production of the microalgae have not been well investigated.

**Results:**

This work demonstrates the feasibility of using outdoor tubular photobioreactors (PBR) for whole-year-round cultivation of a carbohydrate-rich microalga *Scenedesmus obliquus* CNW-N in southern Taiwan. Time-course profile of the carbohydrate content under nitrogen-deficient conditions was monitored to assess the seasonal changes. The optimal CO_2_ fixation rate and carbohydrate productivity were 430.2 mg L^−1^ d^−1^and 111.8 mg L^−1^d^−1^, respectively, which were obtained during the summer time. Under nitrogen starvation, the microalgal biomass can accumulate nearly 45–50% of carbohydrates, mainly composed of glucose that accounted for 70–80% of the total carbohydrates in the microalgal cells. This glucose-rich microalgal biomass is apparently a very suitable carbon source for bioethanol fermentation.

**Conclusion:**

This work shows the feasibility of combining CO_2_ fixation and bioethanol production using microalgae grown in outdoor photobioreactors as feedstock. The understanding of the seasonal changes in the carbohydrate productivity makes this approach more practically viable. The novel strategy proposed in this study could be a promising alternative to the existing technology dealing with CO_2_ mitigation and biofuels production.

## Background

The environment is currently being harmed by the combustion of fossil fuels and the related emissions of CO_2_, which are a significant cause of climate change [1]. Much attention is thus being paid to reducing CO_2_ emissions and developing alternative energy sources using microalgae as a feedstock, due to its higher solar energy yield, high environmental tolerance, and no seasonal limitations [2, 3]. While there is a considerable industrial interest in converting microalgal biomass into biofuels, with biodiesel produced from microalgal oil attracting most of the attention, the production of bioethanol from microalgal carbohydrate is still relatively limited and has received much less attention [4, 5].

Bioethanol is one of the most common liquid biofuels, which has been mostly produced from food crops (e.g., soybean and corn) and lignocellulosic materials (e.g., switchgrass and rice straw) [6], which raises the issue of competition with regard to the food supply, arable land usage, and fresh water, in addition to the high cost associated with conversion of lignocellulosic materials into bioethanol [7]. Microalgae have recently been proposed as a “third generation feedstock” for bioethanol production with the favorable characteristics of a fast growth rate and high CO_2_ fixation ability, while some species can also accumulate large amounts of carbohydrates (mainly, starch and cellulose) that are suitable for bioethanol fermentation [5, 8]. Moreover, the absence of lignin in microalgae also makes the fermentation process much easier when compared with those used with lignocellulosic biomass [5, 9].

To enhance the economic feasibility of microalgae-based bioethanol production, it is necessary to utilize natural sunlight as the solar energy used for the phototrophic microalgae. A growing number of scientists now believe that large-scale outdoor cultivation using sunlight is the only solution for commercial biofuel production [10–12]. However, most microalgal strains cannot be grown reliably outdoors, and the cell growth rate under outdoor conditions is significantly lower than that in the laboratory due to variations in the water temperature and light intensity [12, 13]. To solve the above risks, some studies have demonstrated that using closed photobioreactor (PBR) for microalgae cultivation is an effective and promising method due to its higher light regime, higher culture stability, higher CO_2_ fixation ability, and lower contamination risks compared with open systems (e.g., open pond and raceway pond) [11]. Therefore, to precisely evaluate the yearly outdoor growth performance of strain CNW-N, tubular PBRs, instead of the cheaper open cultivation systems, were performed in this study. To date, there remains limited information regarding the mass production of most microalgae species in outdoor cultivation systems, particularly with regard to long-term outdoor cultivation [14, 15].

**Fig. 1:**
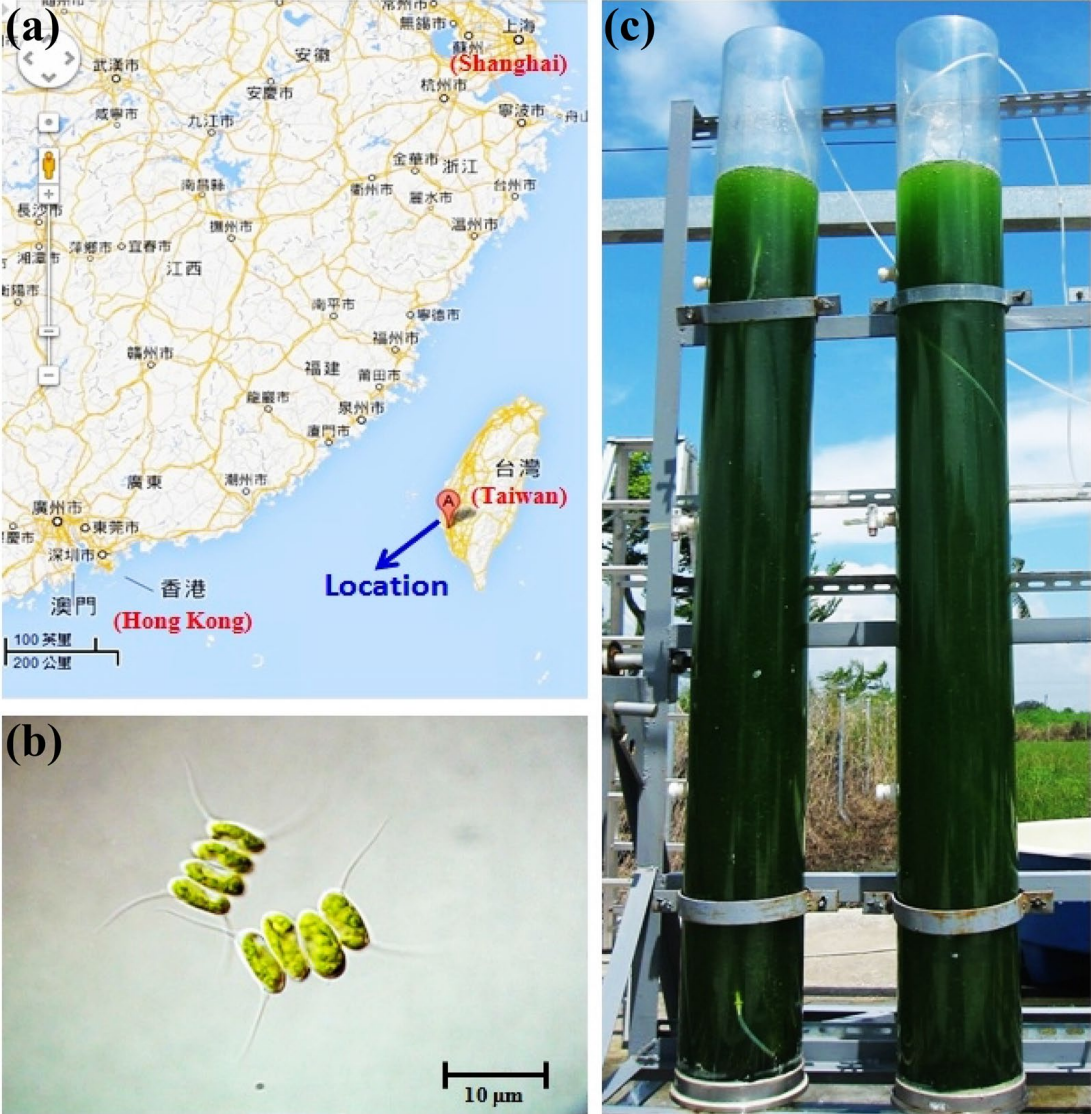
Outdoor cultivation system of large-scale tubular photobioreactors: **a** cultivation location, **b** *S. obliquus* CNW-N, and **c** plastic tubular PBR (60 L)

In recent years, there have been a growing number of reports on the potential for producing biodiesel by the large-scale cultivation of microalgae under outdoor conditions [16–18]. However, only a few studies have reported using microalgae for bioethanol production in the laboratory [2, 7, 9, 19], and no studies have yet focused on the development of an outdoor microalgal-based bioethanol production system. In this work, an indigenous microalga, *Scenedesmus obliquus* CNW-N, with a high cell growth rate and satisfactory carbohydrate content, as demonstrated in our previous research [7, 20, 21], was selected to develop an outdoor microalgal-based bioethanol production system in southern Taiwan (22°99′74.29″N, 120°22′22.30″E) from August 2012 to July 2013 (Fig. 1). The influences of different water temperatures and qualities on the cell growth and CO_2_ fixation rate were first investigated on the laboratory-scale to evaluate the environmental tolerance of *S. obliquus* CNW-N. Furthermore, growth of the algal strain was scaled up to a 60-L tubular PBR with a fixed light intensity of 500 μmol m^−2^s^−1^ and water temperature of 35 °C indoors to determine the growth and carbohydrate accumulation under time-course nitrogen-depleted conditions. Moreover, a year-long outdoor cultivation test was finally conducted to assess the stability of *S. obliquus* CNW-N in long-term batch operations, and then the feasibility of *S. obliquus* CNW-N with regard to serving as a feedstock for bioethanol fermentation was estimated.

## Methods

### The microalga strain and growth medium

The microalga used in this study was a sugar-rich *Scenedesmus obliquus* CNW-N, isolated from freshwater (Niaosung Wetland) located in southern Taiwan [7]. A modified Detmer’s Medium (DM) was used to grow the pure culture of *S. obliquus* CNW-N. The medium consisted of (g L^−1^): Ca(NO_3_)_2_·4H_2_O, 1.00; KH_2_PO_4_, 0.26; MgSO_4_ **·**7H_2_O, 0.55; KCl, 0.25; FeSO_4_ **·**7H_2_O, 0.02; EDTA**·**2Na, 0.2; H_3_BO_3_, 0.0029; ZnCl_2_ 1.1 × 10^−4^; MnCl_2_ **·**4H_2_O, 0.00181; (NH_4_)_6_Mo_7_O_24_ **·**4H_2_O, 1.8 × 10^−5^; CuSO_4_ **·**5H_2_O, 8.0 × 10^−5^.The *S. obliquus* strains were pre-cultured at 28 °C with 2.5% CO_2_ under a light intensity of approximately 60–200 μmol m^−2^ s^−1^ (illuminated by TL5), as measured by a LI-250 Light Meter with a LI-190SA pyranometer sensor (LI-COR, Inc., Lincoln, Nebraska, USA).

### Operation of photobioreactor

The indoor laboratory-scale photobioreactor (PBR) was a 1-L glass vessel illuminated with an external light source (14 W TL5 tungsten filament lamps; Philips Co., Taipei, Taiwan) mounted on both sides. The *S. obliquus* CNW-N strain was pre-cultured and inoculated into the PBR with an inoculum size of 35–40 mg L^−1^. The PBR was operated at 28 °C, pH 6.2, and an agitation rate of 300 rpm. Serving as the sole carbon source, 2.5% CO_2_ was fed at a rate of 0.06 vvm into the culture continuously during the cultivation. The liquid sample was collected from the sealed glass vessel with respect to time to determine microalgae cell concentration, pH, and residual nitrate concentration.

For the scale-up groups, the poly(methyl methacrylate) (PMMA)-made tubular PBRs with working volumes of 60 L (200 cm in height and 20 cm in diameter) were placed outdoors in National Cheng Kung University campus (22°99′74.29″N, 120°22′22.30″E), Tainan, Taiwan, as shown in Fig. 1. Sunlight was the only light supply and the temperature varied naturally depending on the weather situation. In other words, no temperature control system was used. The aeration was 2.5% CO_2_, and the aeration rate was controlled at 0.06 vvm. During the microalgal growth period, liquid samples were collected twice per day from the sealed glass vessel with respect to time to determine microalgal biomass concentration, pH, residual nitrogen concentration, and carbohydrate content/profiles. In addition, the water temperature and light intensity were simultaneously monitored by a LI-250 Light Meter with a LI-190SA pyranometer sensor and a temperature sensor (LI-COR, Inc., Lincoln, Nebraska, USA). The dataset was recorded every 5 min in °C and μmol m^−2^s^−1^, respectively.

### Determination of microalgae cell concentration

The cell concentration in the PBR was determined regularly by measuring the optical density at wavelength 685 nm (denoted as OD_685_) using a UV/VIS spectrophotometer (model U-2001, Hitachi, Tokyo, Japan), after proper dilution with deionized water to give an absorbance range of 0.05–0.9. The dry cell weight (DCW) of the microalgal biomass was obtained by filtering 50 mL aliquots of culture through a cellulose acetate membrane filter (0.45 μm pore size, 47 mm in diameter). Each loaded filter was dried at 105 °C until the weight was invariant. The dry weight of the blank filter was subtracted from that of the loaded filter to obtain the microalgae dry cell weight. The OD_685_ values were converted to biomass concentration via appropriate calibration between OD_685_ and dry cell weight. The conversion factor was determined as 1.0 OD_685_ = 0.43–0.50 g DCW L^−1^.

### Measurement of residual nitrate content

The nitrate concentration in the culture was determined according to the modified method reported in our previous study [21]. A liquid sample from the PBR was filtered with a 0.22-μm pore size filter, and then diluted 20-fold with DI water. The samples were collected and the residual nitrate content was determined according to optical density at wavelength of 220 nm (i.e., OD_220_) using a UV/VIS spectrophotometer (model U-2001, Hitachi, Tokyo, Japan).

### Determination of growth kinetic parameters and CO_2_ fixation rate

The time-course profile of the biomass concentration (X; g L^−1^) was used to calculate the specific growth rate (d^−1^) via drawing of the dry cell weight in logarithmic scale versus time. The biomass productivity (P, mg L^−1^ d^−1^) was calculated based on Eq. 1.

1 $$ P = \frac{{\Delta {\text{X}}}}{{\Delta {\text{t}}}}, $$

where ΔX is the variation of biomass concentration (mg L^−1^) within a cultivation time of Δt (d).

Moreover, according to the mass balance of microalgae, the fixation rate of CO_2_ (mg L^−1^d^−1^) in each PBR was calculated from the relationship between the carbon content and volumetric growth rate of the microalgal cell, as indicated in Eq. 2.

2 $$\begin{aligned} &{\text{CO}}_{ 2} \, {\text{fixation rate (mg L}}^{ - 1} {\text{d}}^{ - 1} ) \\ &\quad = {\text{  P}}{ \times }{\text{C}}_{\text{carbon}} { \times }({\text{M}}_{{{\text{CO}}_{ 2} }} /{\text{M}}_{\text{c}} ), \end{aligned}$$

where P is the biomass productivity (mg L^−1^ d^−1^); C_carbon_ is the carbon content of the biomass (g g^−1^), as determined by an elemental analyzer (Elementar Vario EL III); M_CO2_ is the molar mass of CO_2_; and M_C_ is the molar mass of carbon.

### Determination of the carbohydrate content and profile

The carbohydrate content and profile in the microalgae were determined using the modified quantitative saccharification (QS) method reported by the National Renewable Energy Laboratory (NREL), USA. A small amount of dry algal powder was added to 3 mL 72% (w/w) sulfuric acid and incubated for 0.5 h at 30 °C for the primary hydrolysis. The hydrolysate was then diluted to 4% (w/w) sulfuric acid and incubated for 20 min at 121 °C (sterilization) as the secondary hydrolysis. The supernatant was neutralized and analyzed by high-performance liquid chromatography for sugar assays.

### Acid hydrolysis of microalgae biomass

The acid hydrolysis procedures were based on the method reported by Ho et al. [20]. Sulfuric acid was used as the acidic reagent and 40 g of wet biomass was mixed with the acid to reach at a final concentration of 2.0% (v/v). The resulting slurries were then autoclaved at 121 °C for 20 min. After hydrolysis, the samples were cooled to room temperature, centrifuged at 4 °C and 9000×*g* for 20 min, and the supernatant containing the released sugars was collected as acidic hydrolysate, and the sugar content and composition of this were then measured.

### Operation of separate hydrolysis and fermentation (SHF) for bioethanol production

The efficiency of the fermentative conversion of hydrolyzed microalgae biomass to produce ethanol was examined with the ethanol-producing strain *Zymomonas mobilis* ATCC 29191. The microalgal biomass was first hydrolyzed with 2.0% sulfuric acid based on the procedures described earlier. After acid hydrolysis, the pH of the hydrolysate was adjusted to 6.0 with CaCO_3_, a pH range suitable for ethanol fermentation of *Z. mobilis*. The solid fraction of the hydrolysate was then removed by centrifugation at 10,000 rpm for 10 min. After pre-culture at 30 °C, *Z. mobilis* was centrifuged at 10,000 rpm for 10 min and then inoculated at an inoculum size of 10% (or optical density, OD_600_ = 2.0) to the solution of the hydrolyzed microalgal biomass. Fermentation was carried out at a constant temperature of 30 °C with an agitation of 150 rpm.

### Statistical analysis

Statistical analysis of the data was conducted using the one-way analysis of variance (one-way ANOVA).

## Results and discussion

### Effects of water temperature on the cell growth and CO_2_ fixation rate of *S. obliquus* CNW-N

The growth of microalgae and their cell composition is often significantly influenced by various environmental factors, such as temperature, pH, light intensity, and so on [11, 22]. For outdoor cultivation of photosynthetic microalgae, tolerance to the variable surroundings, especially in temperature and light intensity, is vital, because even a native strain may have difficulties in year-round cultivation during the large changes in weather that occur over time [15, 23]. In order to avoid photo-inhibition outdoors, Feng et al. reported increasing the inoculum size in order to raise the self-shading effect, and thus effectively counteract the extremely high sunlight irradiance [24]. Moreover, the tolerance of the focal species with regard to the local ambient temperature needs to be considered with regard to the outdoor cultivation process, with seasonal changes also taken into account, as this may significantly influence both cell growth and CO_2_ fixation [25].

**Fig. 2:**
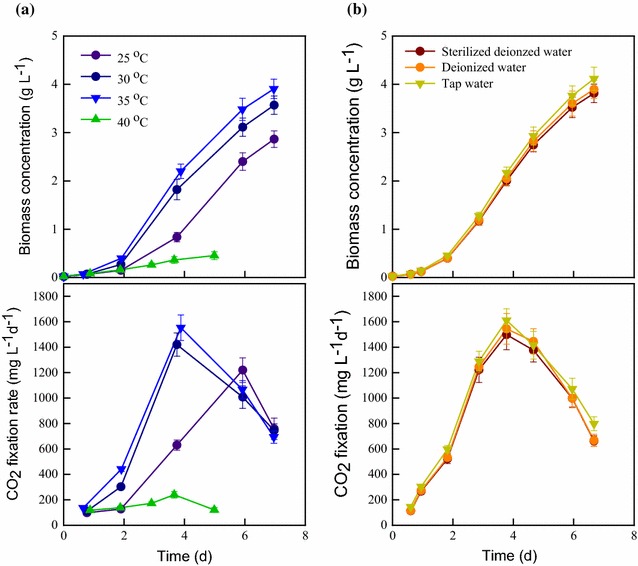
Effects of **a** water temperature and **b** water quality on cell growth and CO_2_ fixation of *S. obliquus* CNW-N grown in the indoor laboratory-scale cultivation system. *Error bars* indicate standard deviation of three replicated experiments. (Light intensity = 220 μmol m^−2^s^−1^; CO_2_ feeding concentration = 2.5%; working volume = 1 L.)

In this study, the indigenous carbohydrate-rich microalga *S. obliquus* CNW-N, with the optimal culture conditions determined in a previous work [26], was grown at various temperatures simulating the different seasons in Taiwan. The cell growth and CO_2_ fixation ability were monitored simultaneously. As shown in Fig. 2a, the highest biomass concentration and CO_2_ fixation rate were 3.90 g L^−1^ and 1066 mg L^−1^ d^−1^ at the water temperature of 35 °C, which are approximately 10 and 30% higher than the values achieved at the water temperatures of 30 and 25 °C, respectively. In addition, Fig. 2b shows that the CNW-N can achieve a high CO_2_ fixation ability when grown in non-sterilized tap water, as well as sterilized deionized water, demonstrating that CNW-N is capable of resisting possible contamination risks from such water resources. Taken together, the results show that a high biomass concentration and a high CO_2_ fixation ability for CNW-N were obtained under the tap water temperature ranges of 25–35 °C, which is similar to the most of the water temperature ranges that occur in southern Taiwan (from February to November), except for the few relatively cold months per year (i.e., December and January). It may thus be feasible to cultivate CNW-N outdoors in southern Taiwan due to its wide temperature tolerance range, and thus it is a good candidate for developing microalgae-based CO_2_ fixation and bioethanol production processes in an outdoor environment with year-round cultivation in this area.

### Effects of nitrogen depletion on the cell growth, carbohydrate accumulation, and CO_2_ fixation ability of *S. obliquus* CNW-N using indoor scale-up PBRs

To enhance the economic feasibility of microalgae-based CO_2_ removal and bioethanol production, in addition to selecting a fast-growing strain with high carbohydrate content, it is also necessary to develop a scaled-up cultivation system with year-round harvesting capabilities [2]. However, not many algal strains are able to be successfully scaled-up from sterilized laboratory-scale to non-sterilized large-scale cultivation, due to the enormous contamination risks associated with the latter. It is thus necessary to evaluate the optimal growth conditions and carbohydrate accumulation ability of the target microalgal strain within a scaled-up cultivation system. In addition, our previous studies have demonstrated that nitrogen depletion can trigger significant carbohydrate accumulation in CNW-N, with a satisfactory cell growth rate, as compared to that seen in related studies [7, 20]. It is thus important to explore how long the microalgal cells should be cultivated under the nitrogen-depleted conditions of a scaled-up cultivation system in order to obtain the optimal carbohydrate production rate and suitable carbohydrate profiles used for the subsequent ethanol fermentation.

**Fig. 3:**
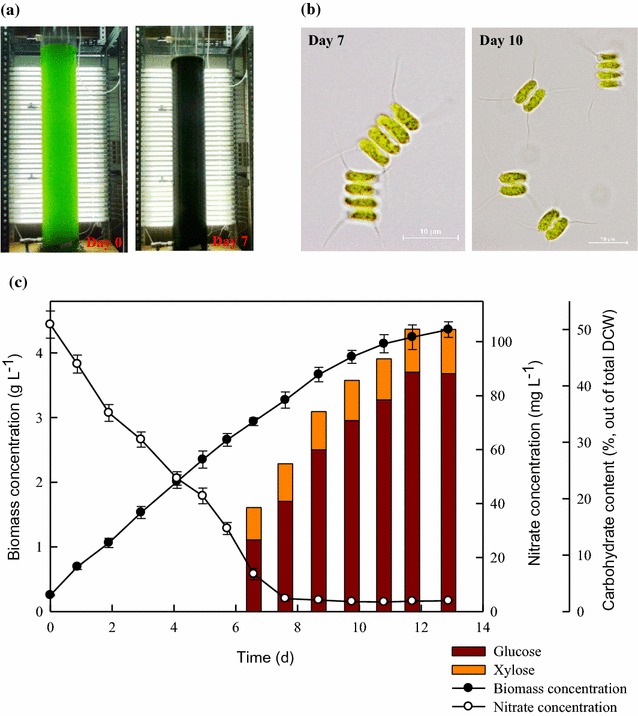
Time-course profiles of biomass concentration, nitrate concentration, carbohydrate content, and carbohydrate profile during the growth of *S. obliquus* CNW-N under indoor large-scale cultivation. *Error bars* indicate standard deviation of three replicated experiments. (Light intensity = 500 μmol m^−2^s^−1^; CO_2_ feeding concentration = 2.5%; working volume = 60 L.) **a** Indoor PBR with external light. **b** Microscopy of S. obliquus CNW-N. **c**Time-course growth of S. obliquus CNW-N under indoor large-scale PBR

In this work, CNW-N was cultivated within a non-sterilized large-scale cultivation system with a continuous external light supply of 500 μmol m^−2^s^−1^ (Fig. 3a), and various growth parameters were monitored, along with the sugar composition. Since there is no circulation system in the PBR, the mixing of the phototrophic culture was achieved with 2.5% CO_2_ aeration (0.06 vvm). As shown in Fig. 3, the cells can grow well in the non-sterilized scaled-up PBR without any contamination. During nitrogen depletion, the carbohydrate content tended to increase significantly along with the duration of nitrogen-depleted conditions, until it reached the highest value of around 50% (Fig. 3c), which is similar to the results found in our previous indoor laboratory-scale system [7]. However, the biomass productivity was 30–40% lower, mainly due to the slower cell growth rate, whereas the accumulation period of carbohydrate was longer, extending from 2 to 4 days [7]. This may be attributed to insufficient light penetration, as other studies have reported that the light distribution inside the PBR will decrease very rapidly [27]. Table 1 shows that after 4 days of nitrogen depletion the biomass productivity, CO_2_ fixation rate, carbohydrate content and carbohydrate productivity were 341.1 mg L^−1^d^−1^, 596.9 mg L^−1^d^−1^, 50.0%, and 170 mg L^−1^d^−1^, respectively. Moreover, in order to enhance the feasibility of ethanol production from microalgal biomass, it is also important to obtain an appropriate carbohydrate composition for further use in ethanol fermentation. As shown in Fig. 3c and Table 1, after 4 days of nitrogen-depleted conditions the glucose content of CNW-N increased significantly from 12.7 to 42.4%, which accounted for the majority of the increase in total carbohydrate content. As such, this strain, mainly composed of glucose (up to 80% of total carbohydrate), seems to be an appropriate candidate for use in ethanol fermentation. These results indicate a highly correlative relationship between the carbohydrate content/profile of strain CNW-N and the duration of the nitrogen depletion period. This suggests that the nitrogen depletion period could be a very important indicator for manipulating the carbohydrate production of CNW-N when used for ethanol fermentation in both laboratory- and large-scale cultivation systems.

**Table 1:** Performance of biomass production, carbohydrate production, and CO_2_ fixation of large-scale batch cultivation of *S. obliquus* CNW-N under indoor conditions with different durations of nitrogen depletion

Cultivation time under nitrogen depletion (d)	Biomass productivity (mg L^−1^d^−1^)	Carbohydrate (glucose) content (%)	Carbohydrate (glucose) productivity (mg L^−1^d^−1^)	CO_2_ fixation rate (mgL^−1^d^−1^)^a^
Nitrogen rich	408.4 ± 20.9	18.4 ± 2.9 (12.7 ± 1.3)	75.1 ± 4.7 (51.8 ± 3.1)	717.7 ± 33.7
0	397.5 ± 13.1	26.2 ± 1.7 (19.5 ± 0.9)	104.2 ± 5.6 (77.6 ± 2.9)	695.7 ± 22.8
1	393.4 ± 15.4	35.5 ± 2.4 (28.7 ± 1.5)	139.5 ± 8.1 (112.8 ± 6.7)	688.5 ± 26.9
2	378.1 ± 9.7	41.0 ± 3.1 (33.9 ± 2.9)	154.9 ± 8.3 (128.0 ± 7.9)	661.7 ± 16.8
3	360.5 ± 10.2	44.8 ± 2.4 (37.5 ± 1.1)	161.4 ± 6.7 (135.3 ± 5.5)	630.8 ± 17.9
4	341.1 ± 11.6	50.0 ± 2.7 (42.4 ± 1.0)	170.7 ± 6.4 (144.7 ± 5.3)	596.9 ± 20.3
5	319.2 ± 13.2	50.0 ± 3.6 (42.1 ± 2.1)	159.6 ± 7.8 (134.6 ± 6.9)	558.5 ± 23.1

Values are the mean ± standard deviation of three replicated experiments. (Light intensity = 500 μmol m^−2^s^−1^; CO_2_ feeding concentration = 2.5%; working volume = 60 L.)

^a^Calculated from the following equation: CO_2_ fixation rate = Biomass productivity (mg L^−1^d^−1^) × C(%) × 44/12

### Effects of large changes in weather on the cell growth, carbohydrate accumulation, and CO_2_ fixation ability of *S. obliquus* CNW-N when grown outdoors in Southern Taiwan

**Fig. 4:**
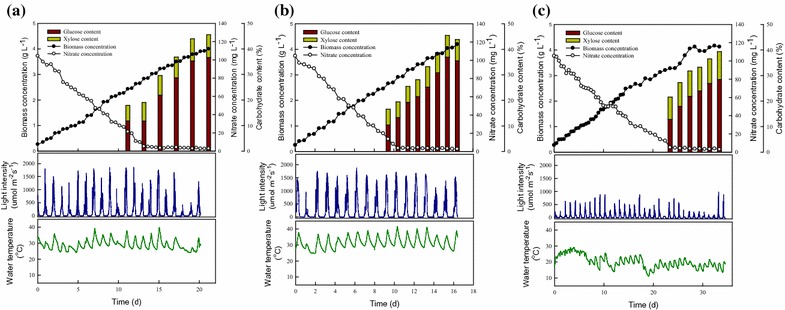
Time-course profiles of biomass concentration, carbohydrate content, carbohydrate profile, water temperature and light intensity of *S. obliquus* CNW-N grown outdoors and under very different weather conditions in southern Taiwan. (CO_2_ feeding concentration = 2.5%; working volume = 60 L.). **a** Rainy (Typhoon) season (Augest 2012). **b** Sunny season (September 2012). **c**Cold season (December 2012)

As noted above, although the growth-related parameters and carbohydrate productivity in a large-scale system are lower than those seen at the lab-scale, the large-scale cultivation of CNW-N without sterilization is still possible in practice. In outdoor cultivation, microalgae not only encounter various contamination risks, but also experience different irradiance and temperature levels day by day due to changes in weather [15, 28, 29]. The photochemical reactions of microalgae are highly affected by irradiance but insensitive to temperature, because there is a biologically imbalanced reaction between the light absorbed through photochemistry versus the energy utilized through metabolism at extremely high irradiance or low temperature conditions [28, 30]. In most algae cultivation systems (especially at the lab scale), irradiance and temperature are maintained at their optimum values, but this greatly increases the production costs. Therefore, the weather tolerance of the target strain and thus the need to avoid the use of an environmental control system (e.g., cooling or heating facilities) are vital for commercial applications. All outdoor cultivations reported in this work were carried out on the National Cheng Kung University campus (22°99′74.29″N, 120°22′22.30″E) in southern Taiwan, from August 2012 to July 2013. The time-course profiles of biomass productivity, carbohydrate content, and carbohydrate productivity of strain CNW-N grown in outdoor PBR under different weather conditions are shown in Fig. 4, with this time period covering the hot, rainy, and cold seasons.

In Taiwan, the climate in the summer is highly changeable due to vigorous southwesterly flow and typhoons. In contrast, the climate in winter is relatively stable but unfavorable for microalgal growth, due to lower irradiance and temperature. It is thus important to examine the influences of large changes in weather on microalgal cultivation and the carbohydrate accumulation rate. As shown in Fig. 4 and Table 2, when the weather turned from rainy (Aug. 2012) to sunny (Sep. 2012) during summer, the average irradiance increased significantly along with a 2.8 °C rise in average water temperature. Under these sunnier conditions, the cultivation time decreased from 21 to 18 days, while the biomass productivity and CO_2_ fixation rate increased from 183.8 to 245.8 mg L^−1^d^−1^ and 321.7–430.2 mg L^−1^d^−1^, respectively. However, the biomass productivity and CO_2_ fixation rate decreased dramatically from 245.8 to 114.9 mg L^−1^d^−1^ and 430.2–201.1 mg L^−1^d^−1^, respectively, when the weather changed from sunny (Sep. 2012) to cold-weather conditions (Dec. 2012) because of a significant drop in both irradiance and temperature. In addition, this study found that the relatively higher temperature and light intensity not only improved microalgae growth, but also enhanced the carbohydrate accumulation rate. The highest carbohydrate productivity of 111.8 mg L^−1^d^−1^ was obtained under the sunny conditions, being around 40% and 2.5-fold higher than the levels seen under the rainy and cold conditions, respectively. Fortunately, although the performance of biomass productivity in December is much lower than that in August and September 2012, the results still show that the large-scale cultivation of CNW-N during the cold season in Taiwan is practical. In addition, the microalgal sugars accumulated by the large-sale cultivation system during different weather conditions were mainly glucose (approximately 75–81% of the total sugars), meaning CNW-N is appropriate for use as a feedstock for ethanol fermentation.

**Table 2:** Comparison of biomass productivity, carbohydrate productivity, and CO_2_ fixation rate of *S. obliquus* CNW-N grown under different weather conditions in outdoor surroundings

Weather condition	Avg. light intensity (μmol m^−2^s^−1^)	Avg. water temperature (^o^C)	Biomass productivity (mg L^−1^d^−1^)	Carbohydrate (glucose) productivity (mg L^−1^d^−1^)	CO_2_ fixation rate (mg L^−1^d^−1^)^a^
Rainy (Typhoon)	182.2	29.1	183.8 ± 18.6	80.8 ± 6.9 (64.6 ± 5.3)	321.7 ± 32.6
Sunny	270.8	31.9	245.8 ± 27.9	111.8 ± 13.6 (90.8 ± 9.9)	430.2 ± 48.8
Cold	52.0	20.2	114.9 ± 20.1	45.0 ± 5.9 (33.9 ± 4.9)	201.1 ± 35.2

Values are the mean ± standard deviation of three replicated experiments. (CO_2_ feeding concentration = 2.5%; working volume = 60 L.)

^a^Calculated from the following equation: CO_2_ fixation rate = Biomass productivity (mg L^−1^d^−1^) × C(%) × 44/12

### Effects of yearly seasonal changes on the cell growth, carbohydrate accumulation, and CO_2_ fixation ability of *S. obliquus* CNW-N when grown outdoors in Southern Taiwan

**Fig. 5:**
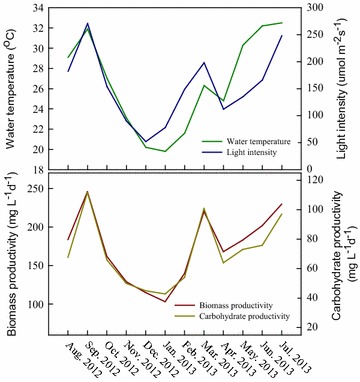
Monthly changes in water temperature, light intensity, biomass productivity, and carbohydrate productivity of *S. obliquus* CNW-N grown under outdoor cultivation in southern Taiwan. (CO_2_ feeding concentration = 2.5%; working volume = 60 L.)

A PBR is one of the most commonly employed devices for outdoor microalgal cultivation due to its higher culture stability and greater volumetric biomass productivity [11, 31]. To date, many studies have examined microalgae-based biofuel production using different PBRs for monthly cultivation [16, 17]. However, very few studies have focused on long-term (e.g., year round) outdoor cultivation of microalgae for biofuel production [15, 32]. Microalgae cultured outdoors can utilize natural sunlight to transform CO_2_ to biomass, and then be transformed into various biofuels. Unfortunately, most microalgal strains are very sensitive to irradiance and temperature, making long-term large-scale outdoor cultivation challenging, even for the local, indigenous strains [32]. Hence, in order to establish commercially viable bioethanol production from microalgae, it is vital to evaluate the stability and feasibility of culturing CNW-N in long-term outdoor testing. In this study, the performance of biomass and carbohydrate productivity over 1 year (Aug 2012–Jul 2013) of batch cultivation in outdoor large-scale cultivation is summarized in Fig. 5. This shows that the trends of water temperature, light intensity, biomass productivity, and carbohydrate productivity were very similar throughout the whole year, revealing relatively low values in the cold month (e.g., Oct–Feb), while reaching relatively high values during spring, summer, and autumn (e.g., Mar–Sep), suggesting that the biomass and carbohydrate production of microalgae is highly related to the outdoor irradiance and temperature. Similar observations were also made by Cuaresma et al. when comparing the differences in cell growth during summer and winter in Huelva, Spain (37°15′N, 6°57′W), demonstrating that the photochemical reactions of microalgae are strongly affected by irradiance and temperature [28]. In particular, a low temperature would rapidly decrease the metabolic activity of microalgae, resulting in lower cell growth and photosynthetic activity, and thus normal irradiance may already be excessive at low temperatures [33]. However, although the biomass and carbohydrate productivity in Dec and Jan are significantly lower than in other months, these results still show the highly reliable, long-term outdoor cultivation of strain CNW-N, as used for ethanol production.

Furthermore, Table 3 shows the performance of biomass productivity, carbohydrate (glucose) productivity, and CO_2_ fixation rate during different seasons in southern Taiwan. The maximum biomass productivity of 205.1 mg L^−1^d^−1^ was obtained in the summer, along with the highest CO_2_ fixation rate of 358.8 mg L^−1^d^−1^ due to the relatively high light intensity and water temperature. This biomass productivity is higher than that obtained in other seasons (in the range of 119.2–190.4 mg L^−1^d^−1^), as well as in most of the related studies examining outdoor cultivation (in the range of 8.7–590.0 mg L^−1^d^−1^), as shown in Table 4. Moreover, since the carbohydrate content did not vary significantly during different seasons (ranging from 39.7 to 42.4%), the highest carbohydrate and glucose productivity (83.9 and 65.6 mg L^−1^d^−1^, respectively) were also obtained in the summer, resulting from the higher biomass productivity. Moreover, the simple sugars derived from the carbohydrate of strain CNW-N are mainly glucose (accounting for 75–81% of the total sugars), which are very suitable ethanol fermentation.

**Table 3:** Comparison of biomass productivity, carbohydrate productivity, and CO_2_ fixation rate of *S. obliquus* CNW-N grown in different seasons in outdoor surroundings

Seasonal condition	Avg. light intensity (μmol m^−2^s^−1^)	Avg. water temperature (^o^C)	Biomass productivity (mg L^−1^d^−1^)	Carbohydrate (glucose) productivity (mg L^−1^d^−1^)	CO_2_ fixation rate (mg L^−1^d^−1^)^a^
Spring (Mar–May)	148.3 ± 44.5	27.1 ± 2.8	190.4 ± 26.8	79.7 ± 19.1 (61.1 ± 16.0)	333.1 ± 46.8
Summer (Jun–Aug)	198.7 ± 43.4	31.3 ± 1.9	205.1 ± 23.1	83.9 ± 9.9 (65.6 ± 7.3)	358.8 ± 40.4
Autumn (Sep–Nov)	171.6 ± 91.7	27.3 ± 4.4	178.9 ± 60.2	75.9 ± 32.1 (60.4 ± 27.0)	313.2 ± 105.4
Winter (Oct–Feb)	93.0 ± 50.2	20.5 ± 1.0	119.2 ± 18.7	47.3 ± 6.0 (35.5 ± 5.2)	208.7 ± 32.7

Values are the mean ± standard deviation of three-month experiments. (CO_2_ feeding concentration = 2.5%; working volume = 60 L.)

^a^Calculated from the following equation: CO_2_ fixation rate = Biomass productivity (mg L^−1^d^−1^) × C(%) × 44/12

**Table 4:** Comparison of the outdoor performance of biomass production, biofuel production, and CO_2_ fixation of *S. obliquus* CNW-N with that obtained from other microalgae species with different PBRs and operation strategies in different locations

Microalgal species	PBR type	Location	Operation mode	Working volume(L)	Biomass productivity (mg L^−1^d^−1^)	Biofuel productivity (mg L^−1^d^−1^)	CO_2_ fixation rate (mg L^−1^d^−1^)	Reference
*C. vulgaris*	Flat-plate airlift	Germany	Batch	30	670	390 (Lipid)	1259.6^a^	[35]
*Chlorella* sp. NJ-18	Airlift	China	Batch	70	91.8	21.9 (Lipid)	172.6^a^	[36]
*Chlorella* sp. NJ-18	Airlift	China	Semi-continuous	70	87.4	24.1 (Lipid)	164.3^a^	[36]
*S. obtusus* XJ-15	Plastic bag	China	Two-stage	70 × 2	86.5	36.4 (Lipid)	170.0	[17]
*B. braunii*	Raceway	India	Batch	80	100	24 (Hydrocarbon)	188^a^	[37]
*S. obtusus* XJ-15	Airlift bag	China	Batch	140	75.6	31.0 (Lipid)	142.1^a^	[38]
*Graesiella* sp. WBG-1	Raceway	China	Batch	40000	8.7	2.9 (Lipid)	16.4^a^	[18]
*N. gaditana Lubián* CCMP 527	Raceway	Spain	Continuous	792	190	30.4 (Lipid)	357.2^a^	[39]
*S. acutus* LB0414	Raceway	USA	Batch	2278	42.9	9.2 (Lipid)	80.7^a^	[12]
*Chlorella* sp.	Plastic bag	Australia	Batch	120	216	74 (Lipid)	406.1^a^	[13]
*T.suecica*	Plastic bag	Australia	Batch	120	179	50 (Lipid)	336.5^a^	[13]
*N. gaditana* Lubián CCMP 527	Tubular	Spain	Continuous	340	590	110 (Lipid)	1109.2^a^	[40]
*Tetraselmis* sp. MUR231	Raceway	Australia	Batch	200	243	85 (Lipid)	456.8^a^	[41]
*Chlorella* sp. CH2	Green Wall Panel	Italy	Batch	10	270	120 (Lipid)	507.6^a^	[42]
*Chlorella* sp.	Tubular	China	Batch	70	154.5	33.7 (Lipid)	290.5^a^	[43]
*Nannochloropsis* sp. F&M-M24	Flat-plate	Italy	Two-stage	110	300	204 (Lipid)	564^a^	[23]
*C. zofingiensis*	Flat-plate	China	Batch	60	58.4	22.3 (Lipid)	109.8^a^	[16]
*S. obliquus* CNW-N (Summer)	Tubular	Taiwan	Batch	60	205.1	83.9 (Carbohydrate)	358.8^b^	This study
*S. obliquus* CNW-N (Winter)	Tubular	Taiwan	Batch	60	119.2	47.3(Carbohydrate)	208.7^b^	This study

^a^ Calculated from the following equation: CO_2_ fixation rate = Biomass productivity (mg L^−1^d^−1^) × 1.88 [3]

^b^ Calculated from the following equation: CO_2_ fixation rate = Biomass productivity (mg L^−1^d^−1^) × C(%) × 44/12

### Bioethanol production using acid-hydrolyzed wet biomass of *S. obliquus* CNW-N grown outdoors via the SHF process

**Fig. 6:**
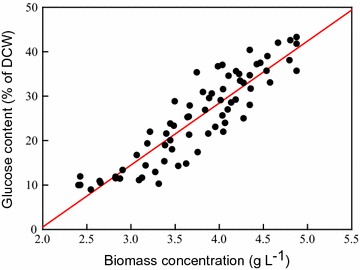
Increases in the glucose content of *S. obliquus* CNW-N with harvested dried biomass. (Linear regression of 71 batch samples from August 2012 to July 2013, R^2^ = 0.82.)

To further evaluate the feasibility of bioethanol production from outdoor cultivation of strain CNW-N, the linear regression between biomass concentration and glucose content was obtained, as depicted in Fig. 6. A higher biomass concentration typically resulted in greater glucose accumulation because of longer periods of nitrogen depletion, which is consistent with the general theory regarding glucose accumulation in microalgae during nitrogen-depleted conditions, as proven in various related reports [7, 34]. It was also clearly demonstrated that the glucose content accumulated to over 30% when the biomass concentration of the microalgae culture reached around 4.1–4.2 g L^−1^, which was cultivated for 15–28 days depending on different weather conditions. Therefore, the biomass concentration is a simple but important indicator to decide the appropriate harvesting time when using a large-scale outdoor cultivation system.

**Fig. 7:**
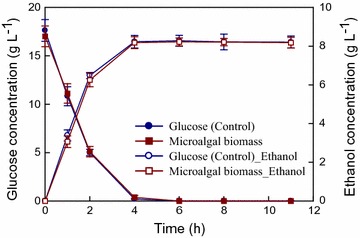
The ethanol production performance via the SHF process using wet biomass from outdoor cultivation as the feedstock. *Error bars* indicate standard deviation of three replicated experiments. (Control means that ethanol fermentation was carried out using pure glucose at the same glucose concentration as the microalgae hydrolysate.)

In addition, the fermentation of hydrolyzed microalgae biomass to ethanol was investigated using the ethanol-producing strain *Z. mobilis* ATCC29191 with the separate hydrolysis and fermentation (SHF) process demonstrated in our previous study [20]. The CNW-N biomass cultivated using the outdoor cultivation system had a glucose content of 42–44% (per dry biomass weight) in this study. This biomass was then hydrolyzed using the optimal acid hydrolysis conditions indicated in our previous report (40 g L^−1^ wet biomass with 2% sulfuric acid) [20], leading to an initial glucose concentration of about 15.9–18.1 g L^−1^ for wet biomass. The *Z. mobilis* cells were inoculated into the microalgal hydrolysate at an initial inoculum size of an optical density of 2.0 (about 0.7 g/L) to carry out ethanol fermentation at 30 °C, and an initial pH of 6.0. Pure glucose was used as the carbon source instead of wet microalgae hydrolysate, but with the same glucose concentration as the control group. As shown in Fig. 7, shortly after the inoculation of *Z. mobilis* cells, the glucose concentration in the groups of pure glucose and wet biomass all dropped significantly, along with a sharp increase in the ethanol concentration. The ethanol production rates obtained from using pure glucose and wet biomass were quite similar, suggesting that the acid hydrolysis process did not produce inhibitory byproducts. Using the wet biomass, a maximum ethanol concentration of 8.18 g L^−1^ and a maximum ethanol yield of 0.205 g ethanol/g biomass were achieved within 4 h of ethanol fermentation. This SHF process achieved nearly 94.1% of the theoretical ethanol yield based on the available glucose content in the microalgal biomass. Therefore, the acid hydrolysis of CNW-N biomass obtained from outdoor cultivation for ethanol production via the SHF process seems to be a promising approach for microalgal-based bioethanol production.

## Conclusions

This work clearly demonstrates that *S. obliquus* CNW-N can adapt to the fluctuating outdoor environments in southern Taiwan, as it was successfully cultivated in large-scale tubular PBRs from August 2012 to July 2013; this study also examined the important parameters of cell growth, CO_2_ fixation rate, and corresponding carbohydrate (glucose) content. The significant influence of light intensity and temperature on cell growth and carbohydrate accumulation under outdoor conditions were demonstrated. The highest biomass productivity, CO_2_ fixation rate, and carbohydrate productivity obtained in this work were 245.8, 430 and 111.8 mg L^−1^d^−1^, respectively (at September 2012), which are better than most of the values reported in recent studies. The main carbohydrate composition was glucose (75–81% of the total carbohydrates), which is very suitable for ethanol fermentation. Therefore, *S. obliquus* CNW-N is a promising candidate for the large-scale, outdoor production of bioethanol feedstock in southern Taiwan. The outdoor open pond/raceway cultivation system together with different cultivation strategies will be examined in the next stage of this project, to find ways to further decrease the cultivation cost and increase the biomass/carbohydrate production potential of *S. obliquus* CNW-N.

## Abbreviations

PBR: photobioreactor
SHF: separate hydrolysis and fermentation
PMMA: poly(methyl methacrylate)
OD: optical density
DCW: dry cell weight
HPLC: high-performance liquid chromatography
QS: quantitative saccharification
NREL: National Renewable Energy Laboratory
